# Registration system of cloud campus by using android smart tablet

**DOI:** 10.1186/2193-1801-3-761

**Published:** 2014-12-22

**Authors:** Shin Kamada, Takumi Ichimura, Tetsuya Shigeyasu, Yasuhiko Takemoto

**Affiliations:** Community Liaison Center, Prefectural University of Hiroshima, 1-1-71, Ujina-Higashi, Minami-ku, 734–8558 Hiroshima, Japan; Faculty of Management and Information Systems, 1-1-71, Ujina-Higashi, Minami-ku, 734–8558 Hiroshima, Japan

**Keywords:** Cloud campus system, Open source software, Attendance management system, Near filed communication, Android application, PC linux, NFC registration system

## Abstract

Near Field Communication (NFC) standard covers communication protocols and data exchange formats. NFC technology is one of radio-frequency identification (RFID) standards. In Japan, Felica card is a popular way to identify the unique ID. We developed the attendance management system (AMS) as the Android application which works in the smart tablet with NFC. Generally, the AMS in the university is fixed to the wall and each student touches or slides his/her own card to the dedicated equipment. Because a teacher can use his/her own smart tablet and/or smartphone, the attendance records are viewed anytime and anywhere. Moreover, we developed the collecting system between PC and some tablets by using Android beam. Any personal data are encrypted and the file can be transferred over the NFC Bluetooth Handover between PC Linux and smart tablet. By the mining of the collected records, early discovery for chronic non-attenders are extracted in educational affairs section. In this paper, a registration system on the cloud campus system by using the personal smartphone with NFC is developed. The system enables to introduce the university courses that are open to the general public.

**Electronic supplementary material:**

The online version of this article (doi:10.1186/2193-1801-3-761) contains supplementary material, which is available to authorized users.

## Introduction

Recently the university is required to check exactly the record for attendance of their student, because Ministry of Education, Culture, Sports, Science and Technology in Japan imposes the ratio of attendance as approving credits. The chronic truancy students receive fewer hours of instruction and tend to cut classes. We know the chronic absenteeism is not the main reason of academic failure, but it remains one of many reasons of leaving school (Rothman^2001^). The relationship between student and university may be important factor for the problem of Chronic absenteeism, truancy and academic failure.

According to the change of social environment such as more relaxed education policy in Ministry of Education, Culture, Sports, Science, and Technology, it is required that university needs to be more student-centered and supportive of students with different needs.

Recently, the attendance management system (AMS) with Radio Frequency IDentification (RFID) technology has been developed as a part of Smart University (Mitchell et al.^2003^, Ueki et al.^2005^), which develops the educational infrastructure using high technologies, such as ICT. However, the reader/writer for Felica is too expensive to build the AMS. Furthermore, the reader/writer is set at each lecture room and the teacher cannot witness the student’s authentication of the ID card, so that, we may meet that one of friends instead of the person in question can answer the roll call for an absentee.

Near Field Communication (NFC) is one of international wireless communication protocols and data format in the communication. For example, the RFID standard is well known wireless data communication method. In Japan, Felica card is a popular way to identify the unique ID. NFC technology includes not only Felica but other type of IC chips. The Android OS 2.3 and the later can provide access to NFC functionality library. Although the Nexus series such as Nexus4, 7(2013) & 10 are not compatible with Mifare classic tags. However, they can detect the UID code of a tag. If we develop an application which has the ability to just detect a tag and read it’s UID code, and then launch settings/profiles/etc then we can still use Mifare Classic tags. Accordingly, we developed AMS Android application by using Nexus series with NFC (Ichimura and Kamada^2013^, Kamada and Ichimura2014a,b). Because Nexus 7 is a low cost smart tablet, a teacher can determine to use familiarly. Any teacher, who calls the roll at the class, can free from stress before the lecture. Especially, this paper describes the method of early discovery for chronic non-attenders by using NFC Bluetooth Handover between PC Linux and Android. The attendant records can be collected from different Nexus 7 and then the data are merged into a SQLite file. The system reports a summary sheet to operate with the trunk system in educational affairs section.

Our university has been developing the cloud campus service system to create or produce the information flow as a bridge between our university and the local community. The cloud service system provides to permit an efficient flow of knowledge and intellectual ability between communication network as flow of electronic information and as flow of interpersonal relationship. The system consists of Social Network Service (Kazu-fuku^2011^), File Sharing Service (Karlitschek et al.^2012^), Video Streaming Service (ViMP GmbH^2010^), and Video Conference Service (Wagner et al.^2006^).

The section Near field communication describes the RFID and NFC specification. In the section NFC attendance management system, the functions of our developed NFC Attendance Management System will be described. Also, NFC Bluetooth Handover between PC Linux and Android will be described. The section Cloud Campus System explains our developed cloud system with some kinds of open source software. In the section Registration system in smart tablet, we explain the software to register the cloud campus system by using NFC. In the section Conclusion, we describe the operation rules of the registration system to the cloud service system such as privacy protection.

## Near field communication

### Radio frequency identification

RFID is the use of a wireless no-contact system. The part of radio frequency uses electromagnetic fields to transfer data from the specified tag. Some tags require no electric power and are recognized at short ranges through electromagnetic induction.

### Range and distance

**Figure 1:**
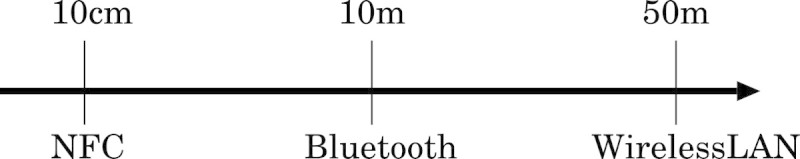
**Communication range.**

Recently, NFC in the RFID tags has attracted a great deal of engineers attention. NFC is a wireless communication technology operating at 13.56 MHz over a short distance of about maximum 10 centimeters. This technology enables the communication among electronic devices to bring into close range of each other, as well as between such devices and conventional contact-less IC cards (SONY Co., Ltd.^2014^). Figure1 shows the communication range of computer network of NFC, Bluetooth, and Wireless LAN RF (RF IDeas Inc.^2014^).

### NFC cards

NFC is derived from communication technology which is specified by the International standard ISO/IEC 18092 (NFCIP-1).

**Figure 2:**
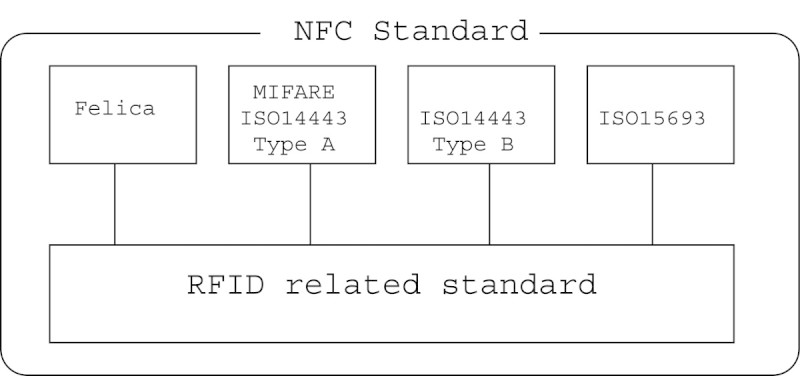
**NFC standard.**

Figure2 shows the concept of NFC. In the NFC Forum specifications, the Type-A and Type-B communication technologies specified in the contact-less IC card international standard ISO/IEC 14443. Their cards are called NFC-A and NFC-B, respectively. The Felica communication technology is called NFC-F and the Japanese Industrial Standard JIS X 6319.4. In Japan, the IC cards have various uses. For example, there are some prepaid or postpaid e-money cards for moving around and shopping.

NFC technologies will be used in various fields of diverse contactless such as healthcare, transportation, information collection, data exchange, seamless payment, store coupons, and so on.

However, the contact-less IC card and its related products have been limited to the cards themselves and reader/writer.

### NFC mode

The specifications of NFC are defined by NFC Forum and the standards for the related descriptions are ISO/IEC 18092 and ISO/IEC 14443-2,3,4, as well as JIS X6319-4 (Sony et al.2004a).

**Figure 3:**
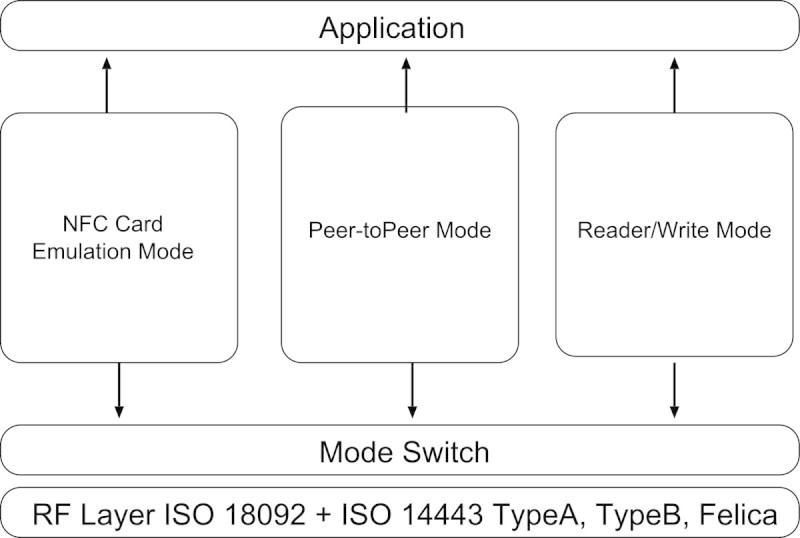
**NFC mode.**

Figure3 shows the 3 modes of NFC: card emulation mode, peer to peer mode, and reader/writer mode (Sony et al.2004b). The card emulation mode is renowned for the general use of contact-less card or tag such as the access control in ticket examination machine, the payments by the electronic money, and the authentication system by the membership card.

The second mode, the peer to peer mode, is the sharing data between 2 or more NFC devices by NFC transmission technique limited within the range of 10 cm. If the size of the sent data is larger, of course, the transfer speed becomes low. In such cases, the data communication is realized by the high speed communication method such as Bluetooth or WiFi after the authentication of each NFC devices or tags. The mechanism is called ‘handover’. The sent data is certificated by the protocol of the second communication method.

The third mode, the reader/write mode, is embedded in the smartphone with NFC device. The traditional mobile phone receives the data through the infrared communication camera. The smartphone without NFC devices recognize the image of barcode captured by the camera. They can translate their codes to the URL of the website or to the alphabetical characters. The mode, for example, can access the information in the tag (seal) such as coupons, product information, and so on, if the smartphone with NFC device taps the tag in their attached paper. The mode realized the easy communication of kinds of information in our daily life.

## NFC attendance management system

In order to be more student-centered and supportive of students with different needs, the project of Smart University (Mitchell et al.^2003^) is underway in some universities.

Recently, the attendance management system (AMS) with RFID technology has been developed as a part of Smart University (Mitchell et al.^2003^), which develops the educational infrastructure using high technologies, such as ICT. However, the reader/writer for Felica is too expensive to build the AMS. Because the developing system with Felica requires the software library provided by the specified maker. The library is prohibited the use under the opensource software. Furthermore, the reader/writer is set at each lecture room and the teacher cannot witness the student’s authentication of the ID card, so that, we may meet that one of friends instead of ther person in question can answer the roll call for an absentee.

NFC reader/writer technology is equipped with the smartphone or smart tablet such as Google Nexus. The smart tablet can easily be carried around, so the student must be checked the attendance in the teacher’s presence. Therefore, the records in the system are correctly and the student becomes an earnest student to attend the lecture for real.

**Figure 4:**
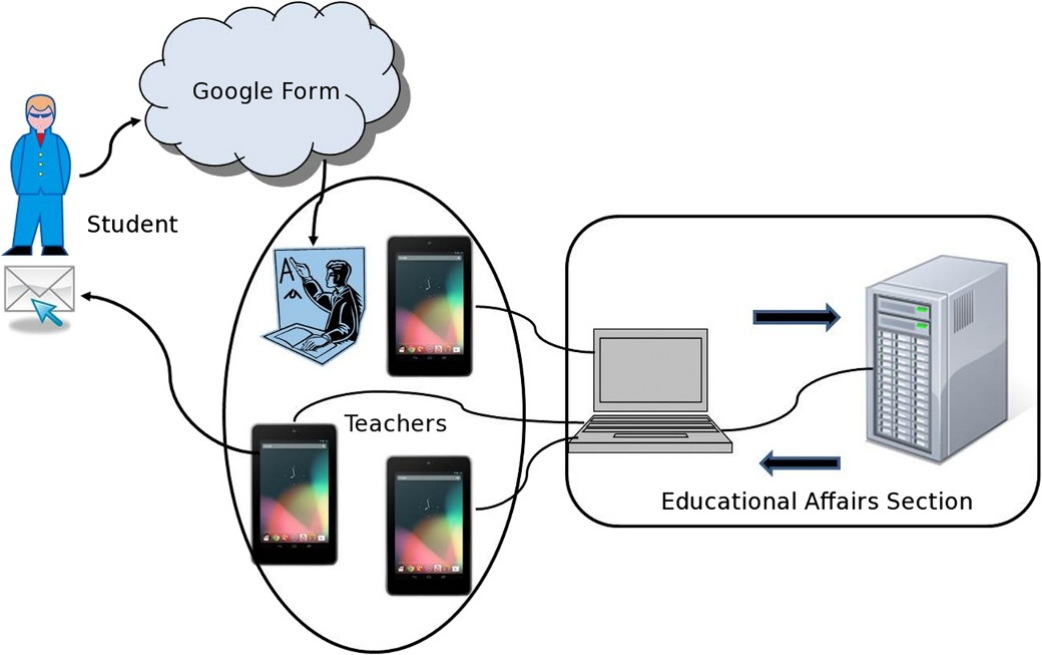
**System overview for AMS.**

Figure4 shows the system overview of our developed AMS system and the system of educational affair’s section. The Nexus part is used to call the roll by teachers. The educational affairs section collects the data related to the absentee by connecting the Nexus to PC after the lecture. The data communication is via Bluetooth connection or the directed connection with microUSB. If the IC card reader/writer is equipped with the PC, data is transmitted automatically after the Peer-to-Peer connection with NFC authentication.

### NFC AMS on smart tablet

This section describes the functions of NFC AMS system on smart tablet (Ichimura and Kamada^2013^;Kamada and Ichimura2014a,b). The system is all-in-one software which has functions to register the information class in charge, to make a list of students, to take attendance, and create follow-up personal message and send it one by one. The teacher can see the reason of the absence through Google Drive. The proving test for the AMS has been approved by the committee of the university education center to promote faculty development activity at Prefectural University of Hiroshima (No: H25FD002) and was implemented from June, 2013 to February, 2014 to the university students who belong to the department of Management Information systems. Written informed consent was obtained from the participant’s guardian/parent/next of kin for the publication of this report and any accompanying images.

**Figure 5:**
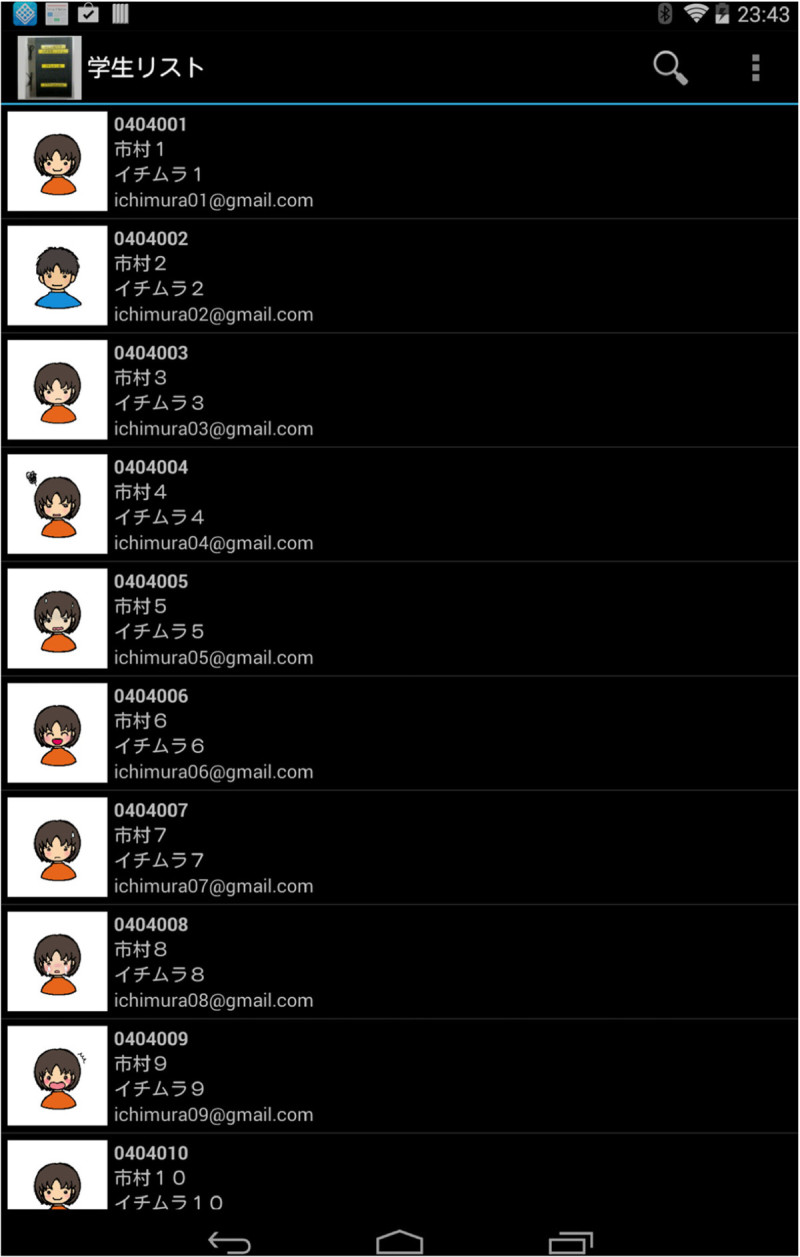
**Student list.**

**Figure 6:**
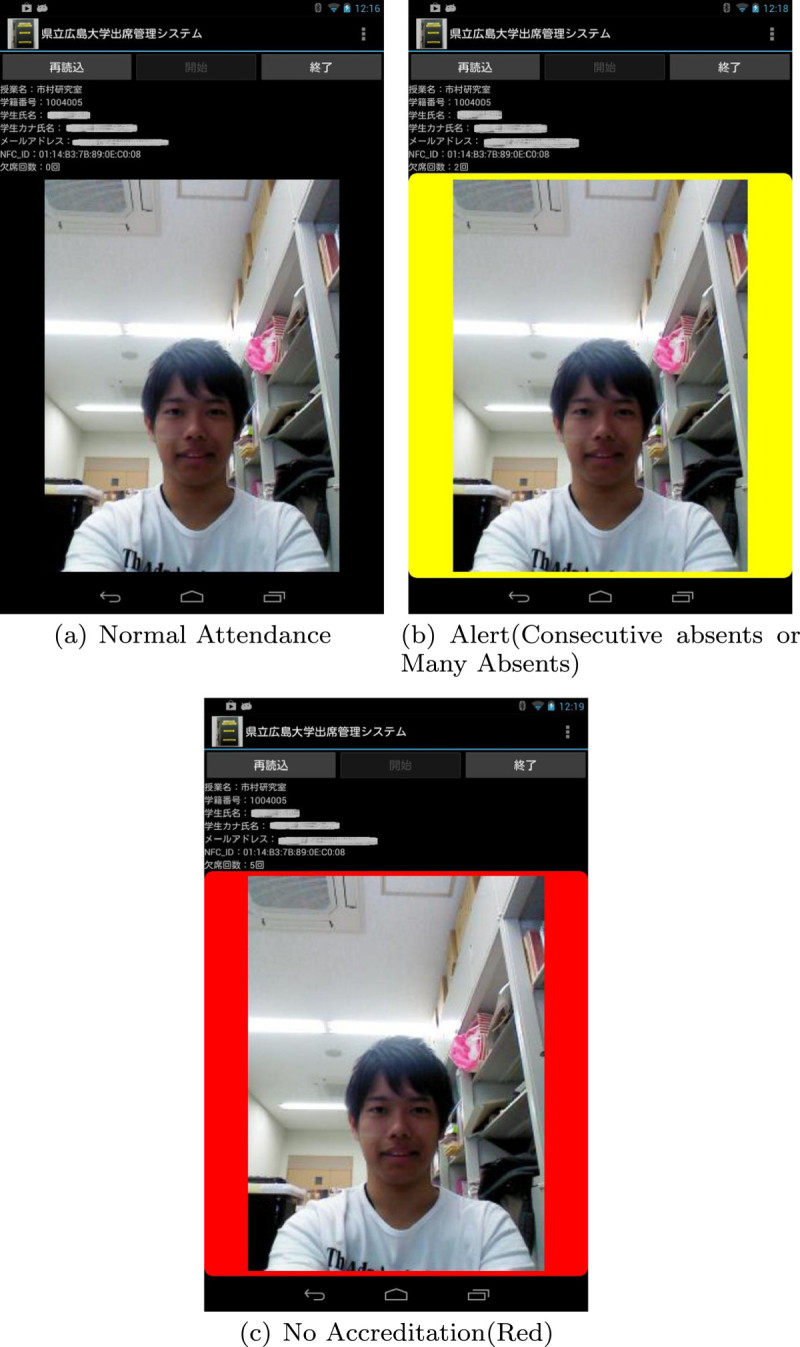
**Alert sysem for absent students. (a)**: Normal attendance. **(b)**: Consecutive absents or many absents (yellow). **(c)**: No accreditation (red).

**Figure 7:**
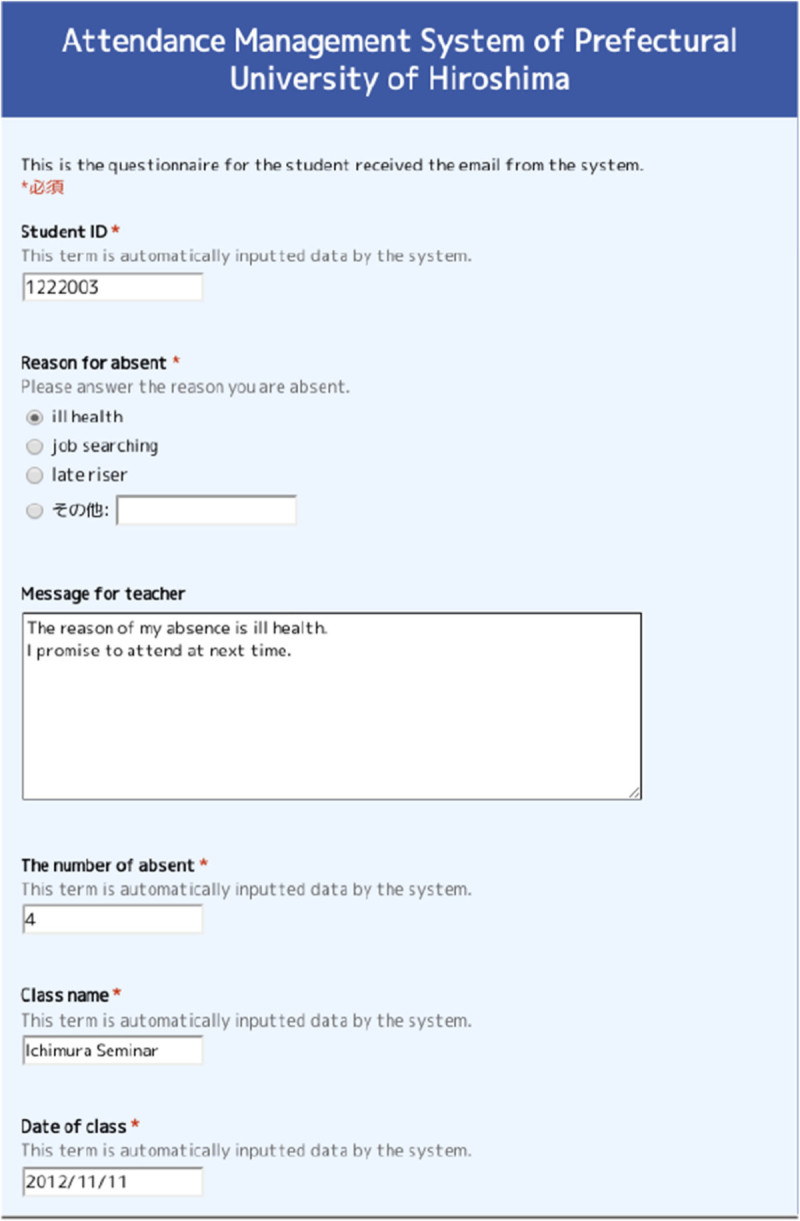
**Google form for absentee.**

**Figure 8:**
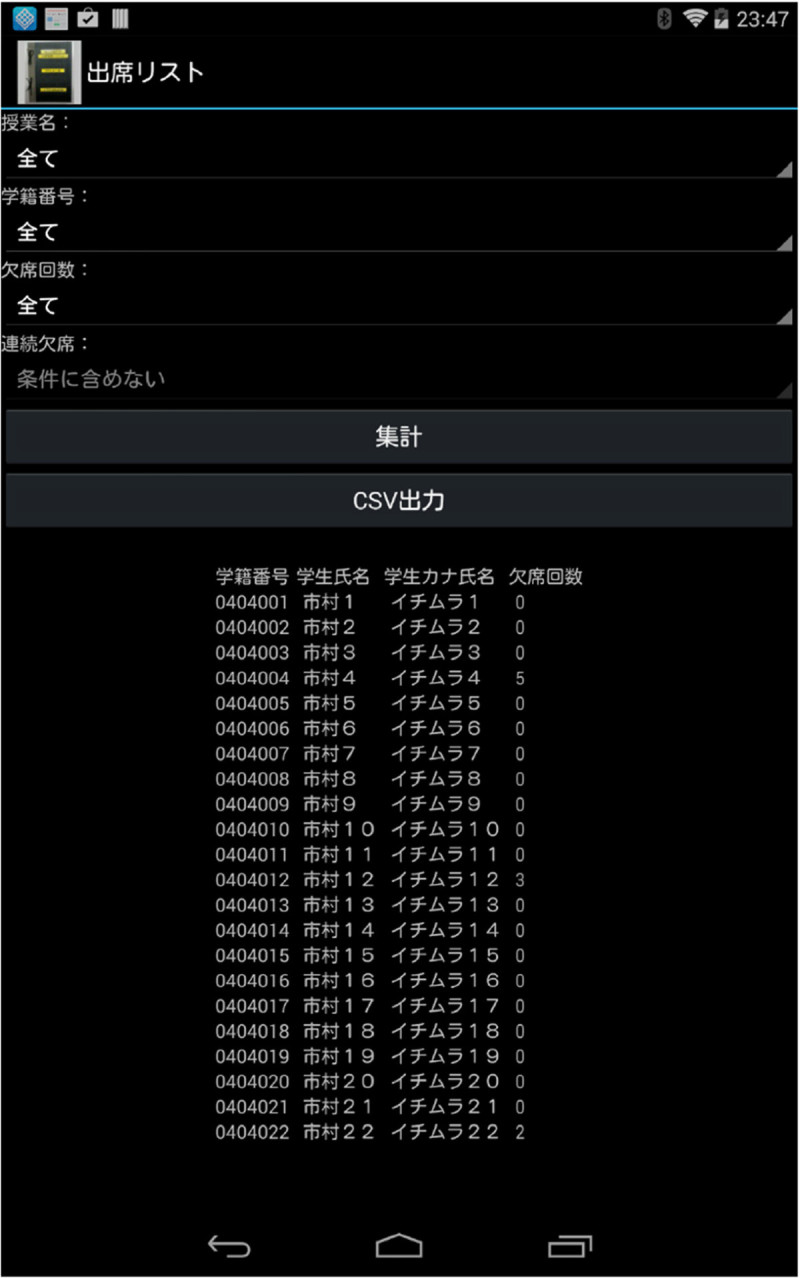
**Tabulation function in the smart tablet.**

- Preparation of class information
  The teacher selects the specified information of class from the educational affair’s section system and makes a CSV file which includes student ID, Name and email address. The AMS inserts the CSV file into SQLite database management system.
- Register the UID of students NFC tag
  Because the smart tablet is equipped with NFC reader/writer, it can read the UID (Unique ID) of NFC card or IDm (Manufacture ID) of Felica card. The user touches the line of student to register NFC card and a face picture student list as shown in Figure5. The numerical value of UID or IDm is scanned and the face of the student is displayed automatically as shown in Figure6, if the card closes to the Nexus. Then, the teacher can recognize the attendance of corresponding student actually.

- Alert to chronic non-attenders
  When the NFC card closes to the Nexus, the system informs to the student who has many absences or is continuation absence, by beep sound or colored display as shown in Figure6(b). Figure6(c) shows the situation of not-approval of credits for the class by regulations.
- Follow-up email
  Follow-up email is sent automatically to the absentee at the class when the teacher finishes the system. The message has the class name, student name, the number of absences, and the specified URL, which is generated individually. The URL is the Google form including the information the class name, student ID, date of lecture and so on as shown in Figure7. The student checks the follow-up email and click the URL, and then he/she can make a confirmation of absence and send the reason for his/her absence and the message to teacher. The teacher can recognize whether the absence of the student is truancy.

- Tabulation on Nexus
  Figure8 shows the tabulation of attendance list on Nexus. The effective function in the class is used, if the student protests the attendance.

- Peer-to-Peer data communication
  After the authentication by NFC, the Peer-to-Peer data communication starts. The AMS data such as the records of attendance on 2 or more Nexus 7 s are merged to be complementary to each other. The function is effective to use in large classroom, because 2 or more Nexus 7 s can be used in a class and the records are synchronized each other.

- Backup and Restore
  The special edition of NFC AMS system can make a backup file in SD card. Of course, it can restore from the data dumped file.

### NFC bluetooth handover between PC linux and android

This section explains our developed connection software to realize the NFC Bluetooth Handover between PC Linux and Android smart tablet. For the developing an application software to read/write NFC tag, the NFC library corresponded to the kind of chip in the hardware of NFC reader/writer should be required.

**Figure 9:**
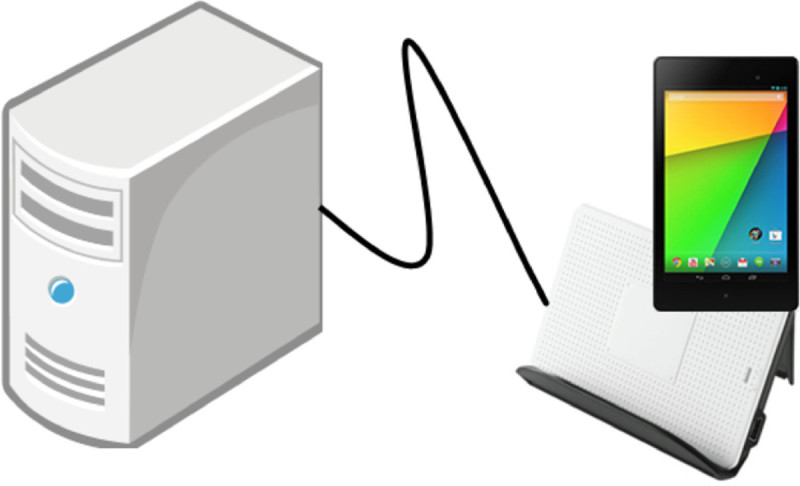
**NFC Bluetooth handover between PC Linux and android tablet.**

We developed the application driver by using ‘Linux-NFC (Ortiz et al.^2012^)’ or ‘Romuald Conty^2009^’ because the chip in the ordinary NFC reader/writer is ‘pn533’ in Japan, called ‘RC-S370’. In the case, the use of ‘libnfc’ is an effective developing method. However, the production of RC-S370 has been stopped. Therefore, only next-generation model RC-S380 is available to develop the application driver for the NFC reader/writer. The libnfc for RC-S380 cannot be supplied the NFC library as open source software. As a result, only the nfcpy^2014^ leads to success in data communication between PC Linux and Android smart tablet as shown in Figure9 (See Additional file1).

The NFC Bluetooth Handover is one of methods of data communication between some devices and the method can enable the data communication without pairing of Bluetooth. The method exchanges the Bluetooth Mac address of two devices, both as close to the device each other. After the exchange of Mac address, actual data communication to the corresponded device will be executed. The advantage in the method can be used only the communication between NFC and Bluetooth. Of course, a more secure communication such as encrypted data will be required. However, the distance between the PC and the smart tablet in the system is limited within the small-scale size. Therefore, the reasons for no-encrypted data communication are as follows. 1) NFC communication is performed by an user’s explicit action such as the touch of cards. 2)The decryption for the encrypted data with the different encryption library will occur the error. Especially, the encryption/decryption system should use the same encryption algorithm, however, the library of Linux Java is not same as that of Android system. For the encryption of different devices, we should adjust some parameters of encryption algorithm in each device. The encrypted data communication has been succeeded, but the time till the end of file takes a long time.

The teachers can receive the list of student data from PC-Linux and vice versa by using the developed data communication system between PC Linux and Android tablet. Therefore, we can find the students with high absent rate and/or find the continuance of the absent students. If a student absents on every Thursday, he/she works at a part-time job on that day. If the other students absent at any first period classes, they oversleep every day. We can perceive the appearance of student who tends to be absent from school. He/she has the possibility for the school refusal.

The relationship between student and university may be important factor for the problem of Chronic absenteeism, truancy and academic failure. According to the change of social environment, it is required that university needs to be more student-centered and support of students with different demands. The NFC Attendance Management System can check the student’s attendance. We are going to mining the characteristics patterns of student behaviors from the collected data. Moreover, the teacher can form a relationship between teacher and student via the system.

## Cloud campus system

A cloud system is categorized into the three following types:

- SaaS (Software as a Service)
  Software as a Service (SaaS) is a software distribution model. In the model, the software applications are not installed in the user’s computer. They are provided by the vendor or service provider and the users can utilized the function of them through Internet.

- PaaS (Platform as a Service)
  Platform as a Service (PaaS) provides the hardware, operating system and network to execute the software application by SaaS.

- IaaS (Infrastructure as a Service)
  Infrastructure as a Service (IaaS) provides the infrastructure for constituting systems, such as CPU, storage, OS, middleware, and so on by using virtualization technology through Internet.

The “Service” in SaaS is “the function of software”. That is, the service offers not the software itself but a function in the network. On the contrary, the term ‘SOA (Service Oriented Architecture)’ is a software architecture design on the construction of a grand scale computer system and the service in SOA means “the software parts for realizing service due to business model cases”. That is, it is a business component in the client-server model computers. Many developments in the field of computer architectures become to provide a great benefit in the computing forms and the data transmission speed in the Internet becomes faster for the last decade. Moreover, both virtualization and tools for the use of big data are realized in our daily life. All the advances in the environment has made it easier to serve and manage their own “Service infrastructure”. Therefore, because SaaS is related to PaaS and IaaS, we can summarize the cloud computing as a large category.

In order to use the cloud service, we use the Google cloud platform. However, the server must preserve the intellectual property of the document/contents and the privacy of the users to construct a Safe and Trusted ICT environment. Therefore, our team aims to separate the own documents and contents. To enable a user to comfortably utilize the server, we develop the authentication of the services for the single sign-on (SSO). The SSO provides a property of access control of multiple related, but independent software system.

### Social network service

Recently we are familiar with the communication on the Social Network Service (SNS), which provides us various abilities under the construction of social community such as to communicate with friends, to talk a chat, to share photos, organize the event, and so on.

**Figure 10:**
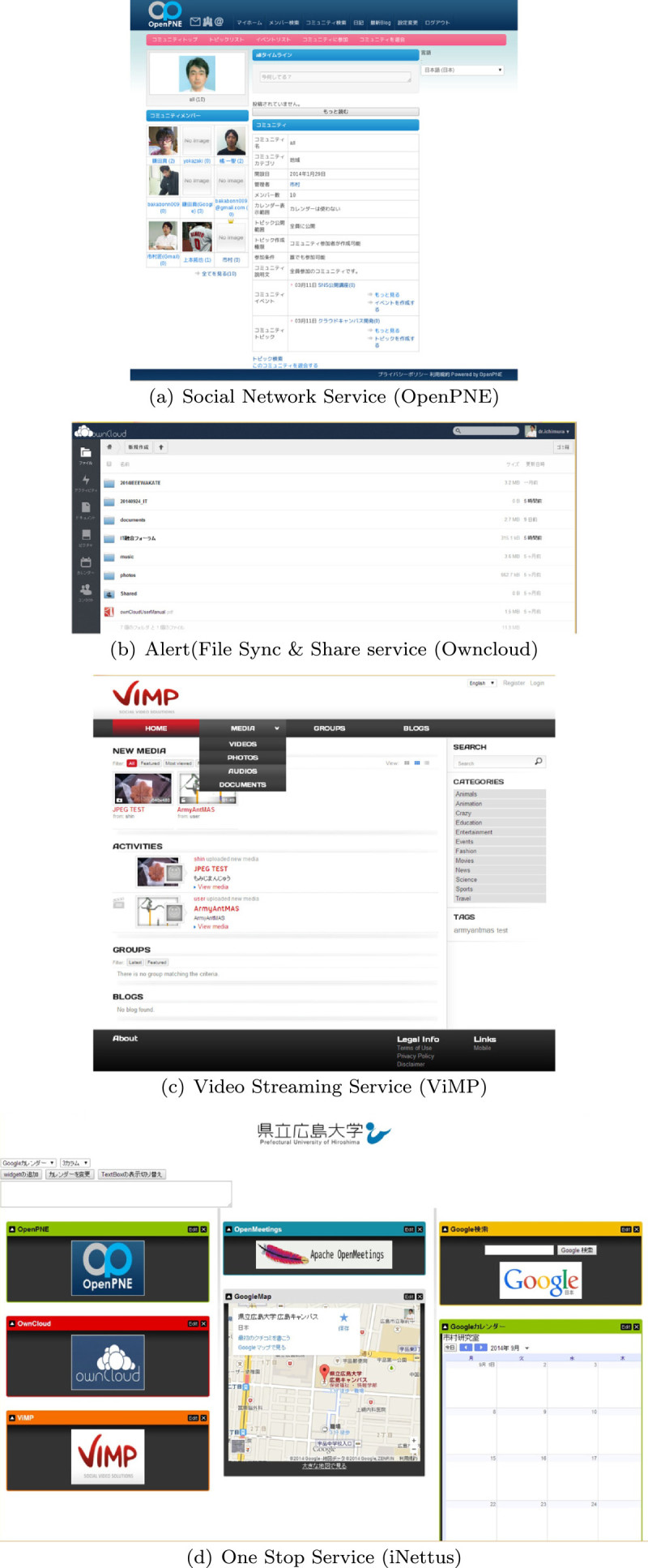
**Cloud campus system with open source software. (a)**: Social network service (OpenPNE) **(b)**: File synchronization & sharing service (Owncloud). **(c)**: Video streaming service (ViMP). **(d)**: Top page for one stop service (iNettus).

We constructed the Social Network Service system by using open source software called “OpenPNE (Kazu-fuku^2011^)”. It provides a full set of functions necessary for SNS including mobile phone. Figure10(a) shows the user’s connection list and time line.

However, the OpenPNE does not have the functions to sync and share the files from all devices such as computers, smartphone, and so on. Especially, the OpenPNE cannot allow to access the specified files to the specified users.

### File sharing service

File Sharing Service (FSS) such as Dropbox, Google Drive, iCloud is becoming the popular service in the Internet. The tool of FSS is equipped with the function of uploading, viewing, and editing the file in easy instruction through the Internet or Smartphone.

The “ownCloud” is an open source file sync and share software and provides a safe, secure, and compliant file synchronization and sharing solution on servers that you control (Karlitschek et al.^2012^). We set the ownCloud to sync and share the file in cloud system.

### Video streaming service

Streaming video distribution system changes the education environment drastically, because the video or the movies can be easily understood, even if we face the difficulty for the reading of the instruction manuals.

The Video Streaming Service such as YouTube is required to obtain educational effects such that a user voluntarily tries to obtain knowledge. Therefore, we use the ViMP Community Edition (ViMP GmbH^2010^) as an open source software.

### Video conference service

For the reason of security, the video conference systems such as Skype are prohibited to use by Deep Packet Inspection (Yu et al.^2006^). Therefore, we must construct the service software under the original network configuration. We developed the system for video conference service by using Openmeethings (Wagner et al.^2006^) which can provide not only video conference but also white board, saving the minutes between person to person or in the group conference. The API of Openmeetings is developed in the Red5 Streaming Server.

### Other information service

Although we can use the various web service, the corresponding urls are required to enter the system. Moreover, the user must remember the login ID and the password to authenticate each service. We developed the authentication function to realize the SSO to above web services (Padolsey^2010^). The iNettuts uses java script with jQuery UI library to mimic the iGoogle Interface. Figure10 shows the entrance site of our cloud system.

The user can register the own profile and picture through the smartphone application.

## Registration application to the cloud campus system in the smartphone

Many kinds of university courses that are open to the general public are planned every year. The university course gives an educational opportunity provided by colleges and universities to people who are not enrolled as regular students. In order to participate in the extention lectures of our university, the persons wishing to participate make request by a postal application. However, it is a state of being troublesome. In case of the receiving the application, the staff must pigeonhole the applications.

**Figure 11:**
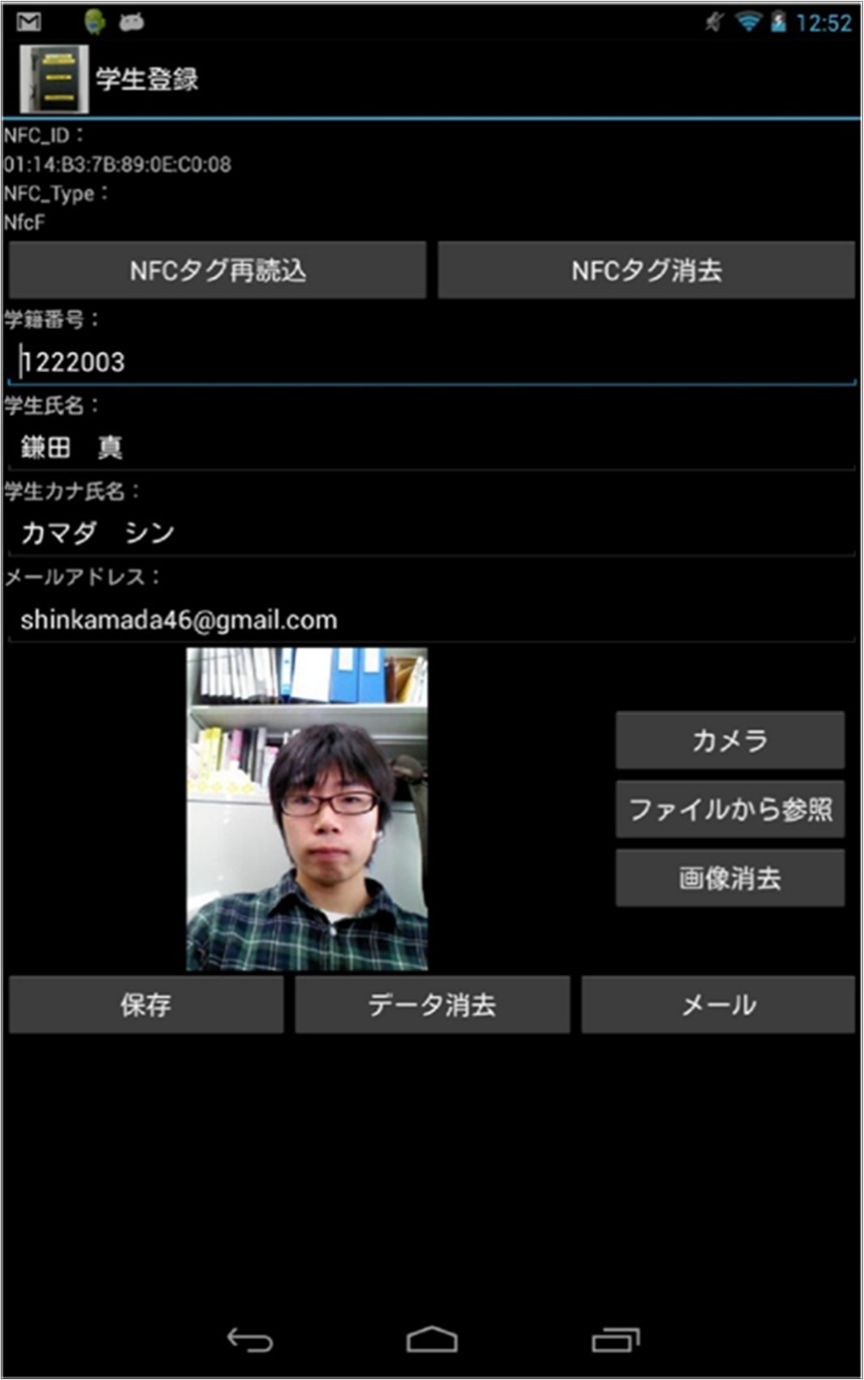
**Registration system.**

Figure11 shows the interface to register the NFC tag in the smartphone/smart tablet such as Nexus. If the user has Android smartphone/smart tablet with NFC reader/writer, he/she registers the personal information and the own NFC tag into the Cloud Campus System. The email address is the already prepared own Google ID. The password is not required to enter, because the password to authenticate the Google ID is used in the Cloud Campus System. The user logins in the SNS service and make a registration for the corresponding lecture, by using the event function of OpenPNE. The user presents the extension lecture and touches the registered NFC tag at the registration desk.

## Conclusion

The relationship between student and university may be important factor for the problem of Chronic absenteeism, truancy and academic failure. According to the change of social environment, it is required that the teacher of university considers that the effective method of more student-centered and supportive of students with different needs.

In this paper, we developed AMS for university with NFC on Nexus. In this system, the teacher can form a relationship between teacher and student, because the teacher can recognize the faces displayed on Nexus when the student touch the ID card to it. Although many investigations are not implemented, absentees at almost classes decreased. We consider that the student has the consciousness of being observed.

Moreover, we have developed the connection software to realize the NFC Bluetooth Handover between PC Linux and Nexus. The software can transfer any encrypted database files between PC Linux and Nexus. So, the attendance situation collected from different Nexus is merged into a SQLite file and then, early discovery for chronic non-attenders are extracted in educational affairs section.

## Electronic supplementary material

Additional file 1: **Movie for the Data Communication PC Linux and Android Tablet.** PCLinux_Android.flv. (ZIP 5 MB)
